# Supplementary material for: X-ray radiation excited ultralong (>20,000 seconds) intrinsic phosphorescence in aluminum nitride single-crystal scintillators
Source: Nat Commun. 2020 Aug 28;11:4351. doi: 10.1038/s41467-020-18221-1 (PMC7455697; doi:10.1038/s41467-020-18221-1)
Supplement: Supplementary file 1 — Supplementary Information [file 41467_2020_18221_MOESM1_ESM.pdf]

Supplementary Information:

X-ray radiation excited ultralong (>20,000 seconds) intrinsic phosphorescence in aluminum nitride single-crystal scintillators

Richeng Lin[1,4], Wei Zheng[1,4,*], Liang Chen[2,4], Yanming Zhu[1], MengXuan Xu[3], Xiaoping Ouyang[2], Feng Huang[1,*]

[1] State Key Laboratory of Optoelectronic Materials and Technologies, School of Materials, Sun Yat-sen University, Guangzhou 510275, China

[2] State Key Laboratory of Intense Pulsed Radiation Simulation and Effect, and Radiation Detection Research Center, Northwest Institute of Nuclear Technology, Xi'an 710024, China

[3] School of Nuclear Science and Technology, Xi'an Jiaotong University, Xi'an 710049, China

[4] These authors contributed equally: Richeng Lin, Wei Zheng, Liang Chen

*e-mail: huangfeng@mail.sysu.edu.cn, zhengw37@mail.sysu.edu.cn

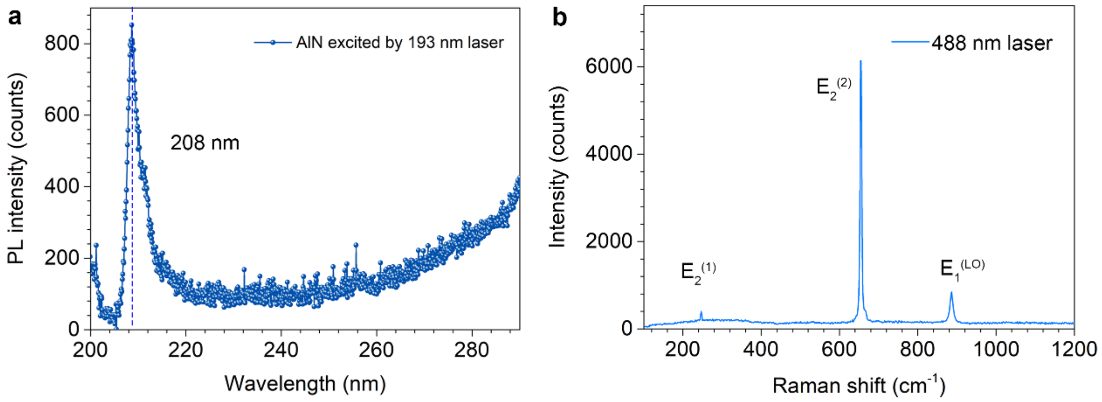

Supplementary Figure 1. Spectroscopy of AlN single crystals (SC). **a**, PL spectrum of AlN SC excited by a 193 nm ArF laser shows an obvious band-edge emission which is located at 208 nm. **b**, Raman spectrum of AlN SC shows vibrational modes of phonons at 248 cm^{-1} ($E_2^{(1)}$), 658 cm^{-1} ($E_2^{(2)}$) and 912 cm^{-1} ($E_1^{(LO)}$) using a 488 nm laser as excitation at room temperature.

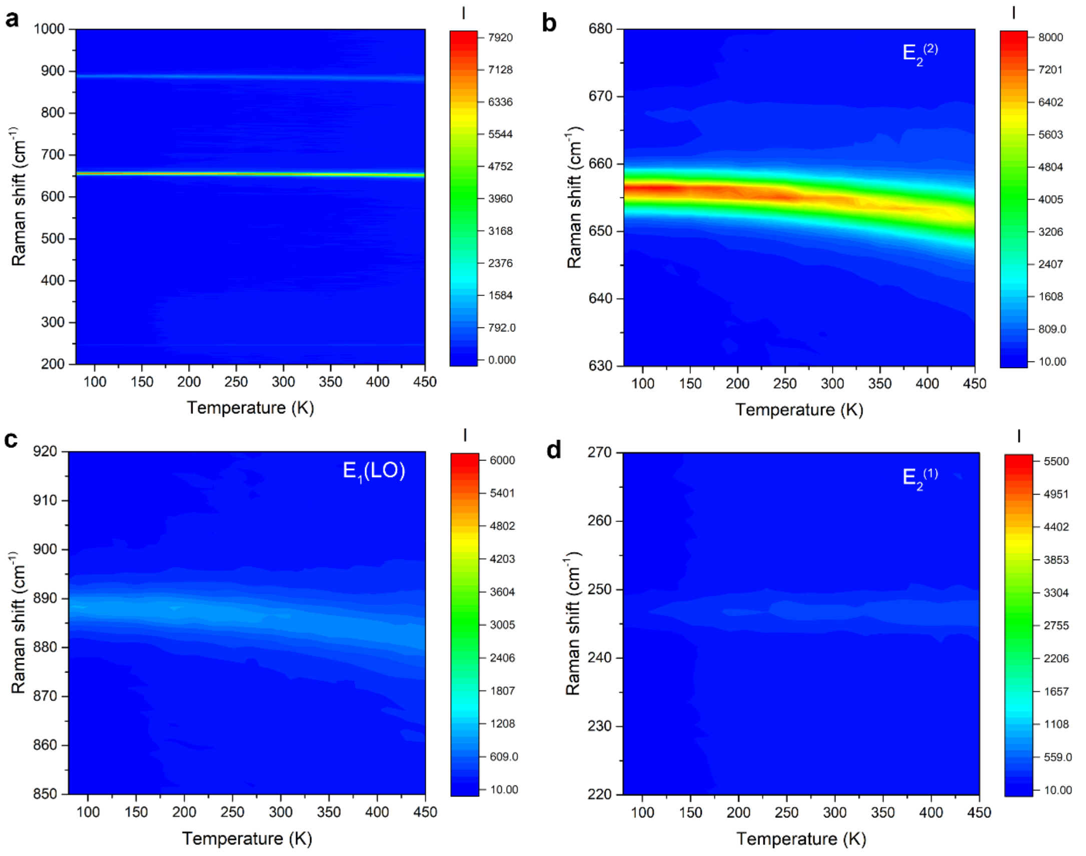

Supplementary Figure 2. Temperature effect of vibrational (phonon) modes. **a**, Temperature-dependent Raman spectrum of the AlN SC. **b**, **c** and **d**, show the detail of $E_2^{(1)}$, $E_2^{(2)}$ and $E_1^{(LO)}$ modes, respectively. The $E_2^{(2)}$ and $E_1^{(LO)}$ modes have similar variation with temperature, showing a broaden of FWHM and a slight red shift. The $E_2^{(1)}$ mode is not detected under low temperature (80 K), and gradually increase with increasing temperature.

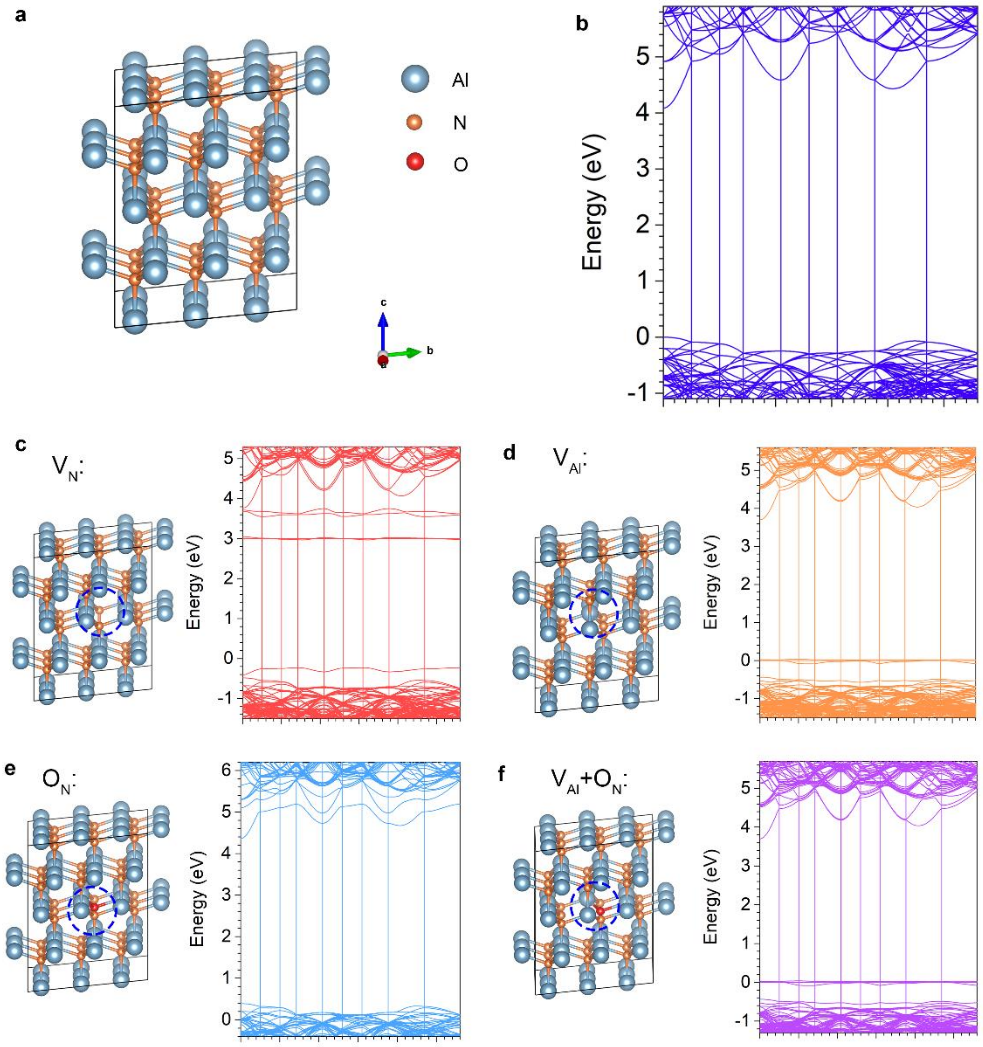

Supplementary Figure 3. Theorical electronic structure of AlN with various point defects. **a**, Crystal structure and supercell of AlN using to theorical calculation. **b**, The calculated electronic structure of original AlN shows a direct bandgap at high-symmetrical G point. The calculated bandgap of original AlN is slightly smaller than experimental bandgap (about 6.0 eV). **c**, Electronic structure of AlN with nitride vacancy (marked by blue dash circle) shows that there are two donor defect energy level which are introduced into the bandgap. Due to the interaction of neighbor atoms, an acceptor defect energy level also is introduced in the bandgap nearing to valence-band maximum. **d**, Observation of the electronic structure of AlN with aluminum vacancy. An obvious acceptor defect energy level is introduced in the bandgap. **e**, Electronic structure of the oxygen substitutional impurity show that no defect energy level is introduced, the energy levels at conduction band minimum are split more obviously. **f**, Couple interaction of oxygen substitutional impurity and aluminum vacancy shows a similar electronic structure to the aluminum vacancy, indicating that the effect of oxygen substitutional impurity is not strong to the electronic structure of AlN. The analysis about electronic structure indicates that the UV (352 nm) and yellow (605 nm) emissions of AlN SCs can be own to the introduction of nitride vacancy.

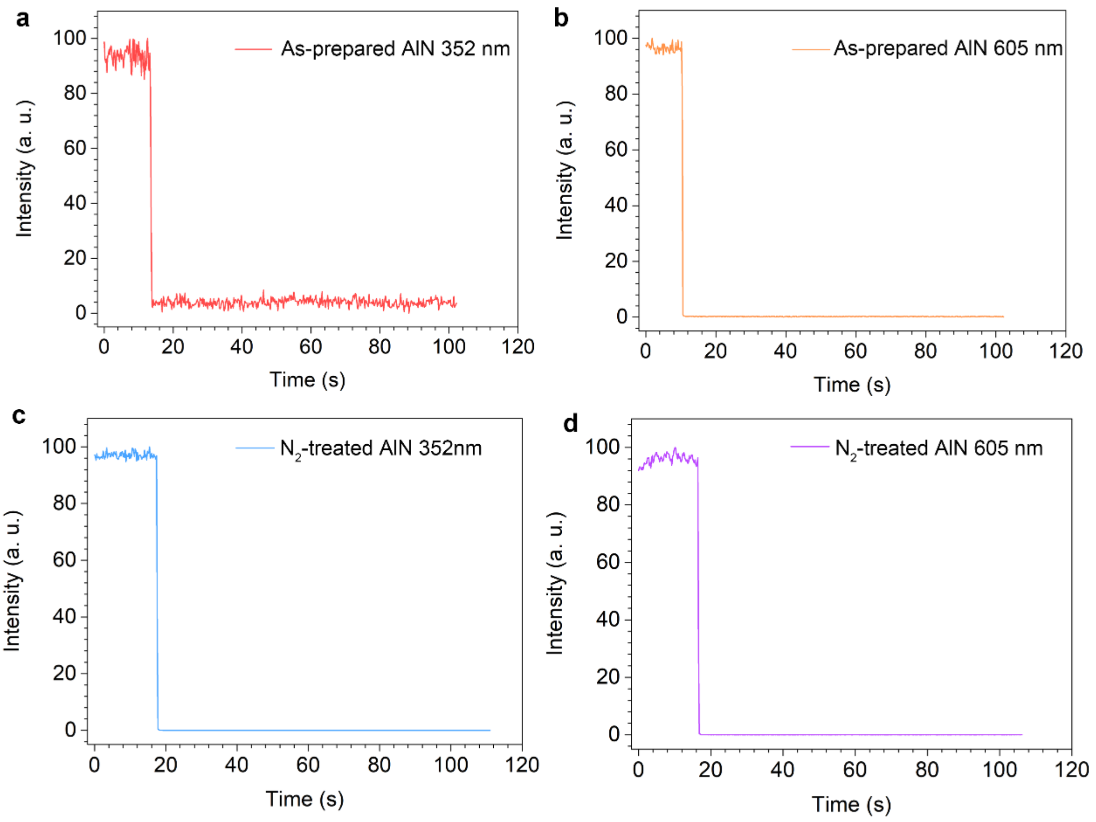

Supplementary Figure 4. Time-dependent PL intensity of AlN SCs under low-energy 266 nm laser excitation. **a** and **b** show the transient PL intensity of the as-prepared AlN SC collected at the wavelength of 352 nm and 605 nm, respectively. **c** and **d** show transient PL intensity that of N_2-treated AlN using high-temperature annealing. Generally, the electronic transition should be followed quantum selection rules. When the excitation stopped, both of the UV emission and yellow emission are fast disappeared, demonstrating that such absorption and radiation transition are strictly followed the quantum selection rules. When under X-ray excitation, the inner-valence-band electrons are excited to high energy states of conduction band, which these transitions may overcome the parity-forbidden rule, while the excitation energy of 266 nm laser (4.66 eV) is relatively lower than that of X-ray radiation. Therefore, the electronic transitions excited by 266 nm laser are followed quantum selection rules, and the radiative transition for light emission is a fast process.

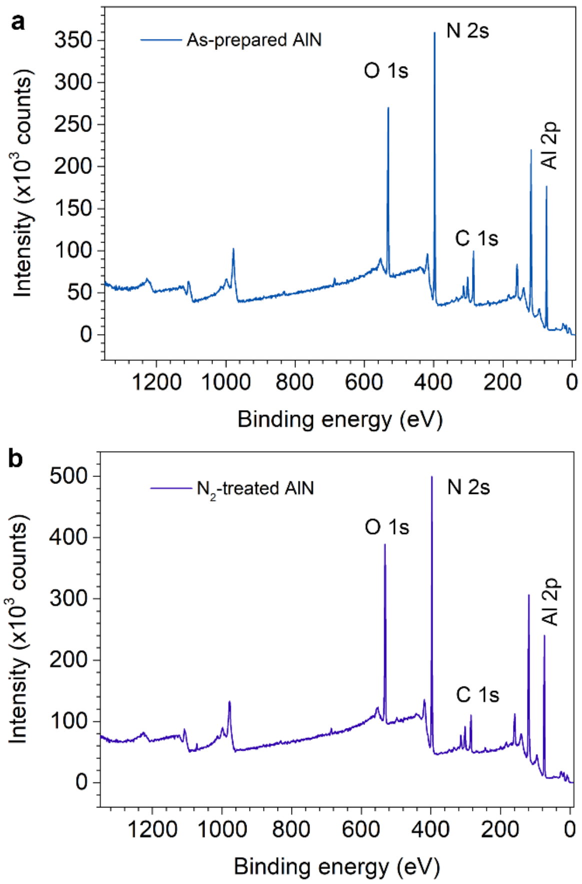

Supplementary Figure 5. X-ray photoelectron spectroscopy (XPS) of the AlN SCs. **a** and **b** show the XPS spectroscopy of as-prepared and N_2-treated AlN, indicating obvious characteristic peak of O 1s, N 2s, C 1s and Al 2p electrons. The detail for elemental analysis is descripted in the main text.
